# Visualizing Mitochondrial F_o_F_1_-ATP Synthase as the Target of the Immunomodulatory Drug Bz-423

**DOI:** 10.3389/fphys.2018.00803

**Published:** 2018-07-04

**Authors:** Ilka Starke, Gary D. Glick, Michael Börsch

**Affiliations:** ^1^Single-Molecule Microscopy Group, Jena University Hospital, Friedrich Schiller University, Jena, Germany; ^2^Institute for Physical Chemistry, Albert Ludwigs University of Freiburg, Freiburg, Germany; ^3^Department of Chemistry, University of Michigan, Ann Arbor, MI, United States; ^4^Abbe Center of Photonics, Friedrich Schiller University, Jena, Germany

**Keywords:** F_o_F_1_-ATP synthase, mitochondria, Bz-423, immunomodulator, drug target, Förster resonance energy transfer FRET, FRET acceptor photobleaching

## Abstract

Targeting the mitochondrial enzyme F_o_F_1_-ATP synthase and modulating its catalytic activities with small molecules is a promising new approach for treatment of autoimmune diseases. The immunomodulatory compound Bz-423 is such a drug that binds to subunit OSCP of the mitochondrial F_o_F_1_-ATP synthase and induces apoptosis *via* increased reactive oxygen production in coupled, actively respiring mitochondria. Here, we review the experimental progress to reveal the binding of Bz-423 to the mitochondrial target and discuss how subunit rotation of F_o_F_1_-ATP synthase is affected by Bz-423. Briefly, we report how Förster resonance energy transfer can be employed to colocalize the enzyme and the fluorescently tagged Bz-423 within the mitochondria of living cells with nanometer resolution.

## Introduction

Cellular processes as metabolism and transport are powered by the universal chemical “energy currency” that is the molecule adenosine triphosphate (ATP). Therefore, millimolar ATP concentrations inside the cell have to be produced and maintained through sequential catalytic reactions by the glycolysis pathway or more efficiently by the F_o_F_1_-ATP synthase as part of the oxidative phosphorylation (OXPHOS) pathway. F_o_F_1_-ATP synthases are working in the plasma membrane of bacteria or in small organelles inside of eukaryotes, i.e., the thylakoid membrane in chloroplasts or the inner mitochondrial membrane. In case of mitochondrial F_o_F_1_-ATP synthase a proton motive force (PMF) comprising a concentration difference of protons across the membrane (ΔpH) plus an electric membrane potential (ΔΨ) is required for ATP synthesis. The PMF is generated by sequential redox processes and associated proton pumping of the enzyme complexes I to IV of the respiratory chain across the inner mitochondrial membrane.

If we consider autoimmune diseases, for example systemic lupus erythematodes, being caused by hyperactivity of pathogenic T cells of the immune system, then controlling their cellular ATP concentration with drugs and reducing their activity could become a promising approach for clinical treatment. Modulating T cell activity temperately could circumvent a complete shut-down of the normal immune function. Therefore, one option would be controlling the PMF by targeting any of the enzyme complexes I to IV of the respiratory chain with inhibitors. *Vice versa*, controlling the efficiency of converting PMF to ATP synthesis by F_o_F_1_-ATP synthase would be a possible approach. This latter process is called uncoupling.

**FIGURE 1:**
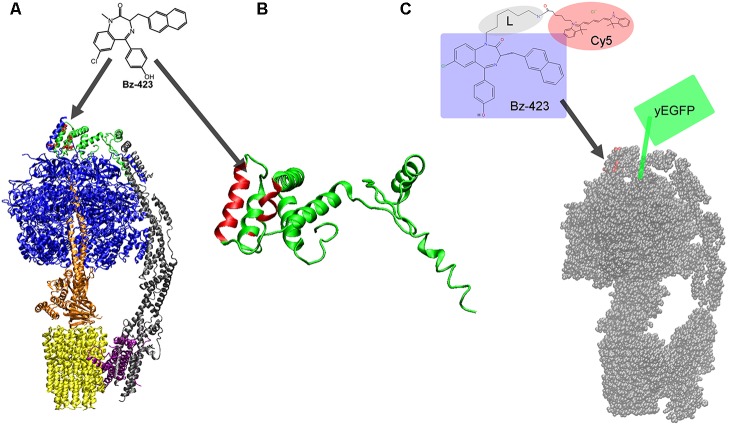
**(A)** Structure of the monomeric mitochondrial F_o_F_1_-ATP synthase from yeast with binding site for Bz-423 (red dots) on subunit OSCP (in green, on the top; from cryoEM data, PDB 6CP3 (Srivastava et al., 2018). Subunits α and β are shown in blue and the rotary subunits γ, δ, and 𝜀 in orange of the F_1_ part. The peripheral static domain consists of subunits *b*, *d*, *F6, f, 8, i, j* (in gray) and OSCP. The proton-translocating subunit *a* is depicted in purple, and the rotor ring of the F_o_ part comprising eight *c* subunits is shown in yellow. **(B)** Structure of OSCP from yeast (PDB 6CP3) with helices highlighted in red (including residues 51, 55, 65, 66, 75, 77, and 92) shown to be involved in Bz-423 binding. **(C)** Bz-423 derivative with the 1,4-benzodiazepine moiety highlighted by the bluish box, a flexible hexyl linker L (gray ellipse) and the FRET acceptor fluorophore Cy5 (red ellipse). Bz-423-Cy5 is expected to bind to OSCP of the yeast mitochondrial F_o_F_1_-ATP synthase (red dots on the top of the monomer of the yeast enzyme, cryoEM data, PDB 6B8H (Guo et al., 2017). The FRET donor yeast-enhanced green fluorescent protein (yEGFP) is fused to the extended C-terminus of the γ subunit (symbolized by the green box).

More than a decade ago, a 1,4-benzodiazepine, Bz-423 (**Figure 1A**), has been found to target lymphoid cells in a murine model of lupus erythematodes (Blatt et al., 2002). Bz-423 specifically induced apoptosis of pathogenic lymphocytes and attenuated disease progression. As a result, the treated mice showed a prolonged survival at the therapeutic dosage without adverse toxicity and with maintained immune function (Bednarski et al., 2003). The mechanism of Bz-423 action was revealed and subsequently the molecular target was identified – the mitochondrial F_o_F_1_-ATP synthase (Johnson et al., 2005). Here we focus on the discovery of the drug binding site and discuss a recent microscopy approach using Förster resonance energy transfer (FRET) that has directly demonstrated the binding of a fluorescent Bz-423 derivative to the mitochondrial enzyme in living cells (Starke et al., 2016).

## Bz-423 Binds to Oscp of Mitochondrial F_o_F_1_-Atp Synthase

Initially, Bz-423 was identified as a potential drug candidate from a library of 1,4-benzodiazepines generated by diversity-oriented chemical synthesis (Blatt et al., 2002). Phenotype screening of Ramos B cells revealed that Bz-423 caused cell shrinkage, nuclear condensation, cytoplasmic vacuolization, membrane blebbing, and DNA fragmentation. Other 1,4-benzodiazepine derivatives were found to selectively target T cells (Francis et al., 2006; Gatza et al., 2011; Wahl et al., 2012; Tkachev et al., 2015). Bz-423 did not strongly bind to the peripheral benzodiazepine receptor. Cytotoxicity of Bz-423 was related to rapidly generated superoxide (O_2_^∙-^) in mitochondria. Superoxide is one of the reactive oxygen species (ROS) that can chemically damage cellular macromolecules at higher concentrations. However, Bz-423 superoxide signaling for induced apoptosis was proven as the underlying mechanism (Blatt et al., 2008, 2009).

In the presence of 1 mM sodium azide, the proapoptotic O_2_^∙-^ generation by Bz-423 was abolished (Blatt et al., 2002). Because sodium azide is an inhibitor of cytochrome c oxidase, i.e., complex IV of the mitochondrial respiratory chain, Bz-423 was proposed to bind to a mitochondrial OXPHOS protein. Binding of Bz-423 did not alter or collapse the electric potential ΔΨ across the inner mitochondrial membrane. The superoxide response by Bz-423 required active mitochondria in state 3 respiration, but not mitochondria in state 4 with minimal respiration and in the absence of ADP. Comparing the superoxide generation mechanism induced by oligomycin that induces a state-3-to-4 transition of mitochondrial respiration (Korshunov et al., 1997) lead to the hypothesis that Bz-423 might cause a state-3-to-4 transition as well and might bind to F_o_F_1_-ATP synthase.

The validation of F_o_F_1_-ATP synthase as the mitochondrial target of Bz-423 was achieved by phage display screening (Johnson et al., 2005). Briefly, the oligomycin sensitivity conferring protein (OSCP) being a subunit of F_o_F_1_-ATP synthase was determined. Subsequently the binding site of Bz-423 was located by NMR spectroscopy using the isolated subunit OSCP in solution (Stelzer et al., 2010). **Figure 1A** shows the structure of the yeast F_o_F_1_-ATP synthase with the highlighted subunit OSCP in green and the amino acid residues involved in binding of Bz-423 as red dots. Accordingly Bz-423 binds to the top of the membrane enzyme at the interface of OSCP with one pair of α, β subunits (blue in **Figure 1A**) of the F_1_ part. Binding of water-soluble Bz-423 analogs to OSCP in a chemical shift perturbation NMR measurement revealed specific residues 51, 55, 65, 66, 75, 77, and 92 that might form a hydrophobic pocket to accommodate the drug (**Figure 1B**). Furthermore, Bz-423 binding resulted in a conformational rearrangement of helices in OSCP and might alter the interaction of OSCP with F_1_ in an allosteric manner (Stelzer et al., 2010).

## Bz-423 Requires Oscp to Modulate F_o_F_1_-ATP Synthase Activity *In Vitro* and in Cells

Binding of Bz-423 to OSCP in the intact F_o_F_1_-ATP synthase inhibited both synthesis and hydrolysis of ATP in isolated sub-mitochondrial particles (SMPs) *in vitro* (Johnson et al., 2005, 2006). Both maximal turnover V_max_ and K_M_ were changed, in contrast to the inhibitor oligomycin which reduced V_max_ only. ATP hydrolysis by the soluble mitochondrial F_1_ part (comprising the blue and orange colored subunits in **Figure 1A**) was reduced but only when F_1_ was assembled with OSCP. The IC_50_ for Bz-423 was about 5 μM. In perfused HEK cells ATP synthesis rates of mitochondria were reduced, with IC_50_ of less than 5 μM for Bz-423. Engineered HEK cells with a specifically reduced content of OSCP by siRNA showed alleviated apoptosis by Bz-423, and the residual amount of cellular OSCP correlated well with the percentage of apoptotic cells (Johnson et al., 2005).

F_o_F_1_-ATP synthase accomplishes ATP synthesis by mechanochemical energy conversion with two rotary subunit motors. The PMF drives the F_o_ motor when protons enter the half-channel of the membrane-embedded *a* subunit (purple in **Figure 1A**) from the intermembrane space, i.e., from below and along the horizontally tilted helices (Allegretti et al., 2015). Binding to a specific residue on one *c* subunit (yellow in **Figure 1A**) compensates electrostatic forces and allows the ring of *c* subunits to rotate one step forward. Rotation of the *c*-ring moves the elastically-coupled attached F_1_ motor (orange in **Figure 1A**) comprising subunits γ, δ, and 𝜀. The F_1_ motor rotates in three 120° steps at high PMF and stops at each of the three catalytic sites where ATP is synthesized in F_1_. These distinct step sizes of the rotary F_o_ and F_1_ motors during ATP synthesis have been measured *in vitro* in single-molecule experiments using bacterial F_o_F_1_-ATP synthases (Diez et al., 2004; Zimmermann et al., 2005; Duser et al., 2009). F_o_F_1_-ATP synthase can also run in reverse by hydrolyzing ATP. ATP hydrolysis has been used to investigate the F_1_ motor in great detail since 20 years (Noji et al., 1997) and revealed torque, elastic domains (Wachter et al., 2011; Sielaff and Borsch, 2013; Martin et al., 2018), substeps (Yasuda et al., 2001; Suzuki et al., 2014), breaks and other motor properties (Yoshida et al., 2001; Junge et al., 2009; Borsch and Duncan, 2013).

Internal subunit rotation with high torque requires a mechanically stable stator counterpart of the enzyme. The static domain of the mitochondrial F_o_F_1_-ATP synthase comprises the six F_1_ subunits α_3_β_3_ (blue in **Figure 1A**), subunits *b*, *d*, *F6, f, 8, i, j* (gray in **Figure 1A**), the *a* subunit (purple in **Figure 1A**) and OSCP (green in **Figure 1A**). Binding of Bz-423 to the interface of OSCP with α_3_β_3_ might weaken the stator/F_1_ assembly. Alternatively, Bz-423 might influence the subtle conformational changes of OSCP bound to the top part of the catalytic α_3_β_3_ subunits and thereby provokes reduced rates of ATP synthesis and hydrolysis. Quantitative enzymatic analysis revealed that Bz-423 is an uncompetitive inhibitor of mitochondrial F_o_F_1_-ATP synthase decreasing V_max_ for ATP synthesis to 50% in the presence of ∼10 μM Bz-423 (Johnson et al., 2006). Inhibition by μM amounts of Bz-423 corresponded to fast off-rates <0.3 s^-1^ of the drug from F_o_F_1_-ATP synthase and a 90% recovery of ATP synthesis rates after 10 s.

## Imaging the Drug and Its Molecular Target in Mitochondria

Localizing a drug at a specific target in life cells can be achieved using fluorescence microscopy with high spatial and temporal resolution as well as single molecule sensitivity. A variety of functional Bz-423 derivatives was synthesized with a flexible linker to rhodamine- or cyanine fluorophores, for example Bz-423-Cy5 (**Figure 1C**). Because μM concentrations of Bz-423 are required to bind to OSCP and to induce apoptosis, direct imaging of the fluorescent drug bound to F_o_F_1_-ATP synthase in the inner mitochondrial membrane is not possible due to a high fluorescent background of unbound Bz-423 throughout the cell. Fast off-rates of Bz-423 and its fluorescent derivatives prevent extensive washing of the cells which is needed to obtain a good imaging contrast. Therefore, confocal microscopy with about 250 nm resolution or superresolution microscopies like structured illumination microscopy (SIM) (Shao et al., 2011) (resolution limit of about 100 nm) or stimulated emission depletion (STED) (Hell, 2007) (resolution limit of about 20 nm) cannot be used to identify bound Bz-423 on OSCP.

Instead, FRET (Förster, 1946, 2012) as a distance measurement approach in the 2 to 9 nm range is applicable. The dipole–dipole interaction of FRET between two nearby fluorophores causes a relative loss of fluorescence intensity of the FRET donor (excited by the laser) and an increase of fluorescence intensity of the FRET acceptor. Thus FRET can be used to relate the spatial position of the fluorescently labeled drug to its cellular target that is tagged with a different fluorophore. Mitochondrial F_o_F_1_-ATP synthase can be tagged with fluorescent proteins, for example at OSCP (Prescott et al., 1997) or at the rotary γ subunit without affecting function (Prescott et al., 2003; Muster et al., 2010; Foertsch et al., 2017). The benefit of using a genetic fusion to the enzyme is a negligible fluorescent background from other parts of the cell than the cristae of the inner mitochondrial membrane.

## Revealing Bz-423-Cy5 Binding to F_o_F_1_-ATP Synthase by Fret Acceptor Photobleaching

**FIGURE 2:**
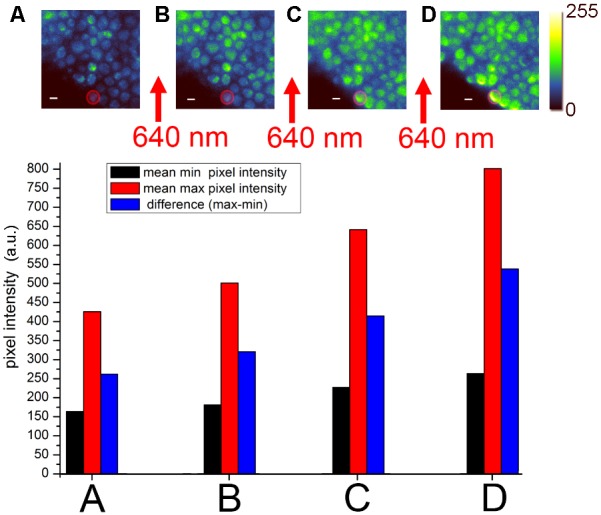
Förster resonance energy transfer (FRET) acceptor photobleaching of Bz-423-Cy5 bound to mitochondrial F_o_F_1_-ATP synthase with yEGFP fusion to the γ subunit in living *Saccharomyces cerevisiae* (Starke et al., 2016). EMCCD-based widefield fluorescence microscopy used laser excitation of yEGFP with 488 nm and fluorescence detection of yEGFP between 500 and 550 nm. **(A–D)** Sequential imaging of yEGFP-tagged F_o_F_1_-ATP synthases with recalculated, false-colored intensities in 8 bit (0–255; upper panel). Between each image, a 30-s high-power laser pulse with 640 nm was applied to partially photobleach the Cy5 chromophore. The individual mean pixel intensities of the highlighted cell [red circle as the region-of-interest (ROI)] are plotted in the histograms in the lower panel as recorded by the EMCCD camera [modified from Starke et al. (2016) with permission].

To detect binding of fluorescent Bz-423 to mitochondrial F_o_F_1_-ATP synthase in living *Saccharomyces cerevisiae* cells, we used a F_o_F_1_-ATP synthase mutant designed by J. Petersen and P. Gräber (I. Starke, Ph.D. thesis, University of Freiburg, 2015). A fusion of the yeast-enhanced green fluorescent protein (yEGFP) linked to the C-terminus of the γ subunit (**Figure 1C**) provided the donor for FRET imaging (**Figure 2**). The fully functional mutant was checked for ATP synthesis, and catalytic rates were also determined *in vitro* after protein purification and reconstitution to liposomes. Staining mitochondria was achieved by incubating the *S. cerevisiae* cells with 4 μm Bz-423-Cy5 (see structure in **Figure 1C**) in the presence of 2% EtOH for 8 h at 28°C. After washing, the bluish cells (**Figure 2**) were imaged immediately on the microscope at 22°C (Starke et al., 2016).

Widefield fluorescence microscopy of the stained yeast showed spherical cells with bright spots suggesting fluorescent F_o_F_1_-ATP synthase in mitochondria (**Figure 2A**). SIM imaging of unstained yeast confirmed that only mitochondria were fluorescent (Starke et al., 2016). Repeated imaging of the same field of view indicated comparable pixel intensities in these cells revealing only minor photobleaching of yEGFP. However, subsequent excitation of the cells with 640 nm at high power photobleached the FRET acceptor Cy5 dye on Bz-423, and the loss of FRET acceptor resulted in an increase of the FRET donor intensity when imaged again with 488 nm (**Figure 2B**). Stepwise photobleaching of Cy5 correlated with a stepwise increase of FRET donor fluorescence (**Figures 2C,D**). Analysis of the intensity increase in individual cells (see histograms of the single cell highlighted by the red circle in **Figures 2A–D**) due to Cy5 photobleaching unequivocally corroborated the binding of Bz-423-Cy5 to a position on F_o_F_1_-ATP synthase only few nm away from the yEGFP chromophore at the extended C-terminus of the γ subunit. A background fluorescence increase (black bars in the histograms) after each 640 nm exposure was subtracted from the increase of maximum fluorescence (red bars) to obtain the effective increase of FRET donor fluorescence (blue bars). Most likely, Bz-423-Cy5 was bound to OSCP.

Förster resonance energy transfer acceptor photobleaching to confirm the molecular target of a drug in living cells is a fluorescence microscopy approach that requires fast imaging capability but not necessarily high photon counts rates per pixel to achieve accurate colocalization. Despite weak (μM) binding affinities of Bz-423-Cy5 and fast exchange of the immunomodulator on OSCP, a significant fraction of bound Bz-423-Cy5 was revealed by FRET. Slow transport of Bz-423 across the membranes into the matrix of mitochondria as the final destination was indicated by long incubation times needed for staining the yeast cells. Accordingly, a significant fraction of Bz-423-Cy5 still remained in the cytosol and outside of the mitochondria during FRET acceptor photobleaching, and did not contribute to apoptotic action. However, slow exchange of photobleached Bz-423-Cy5 into and out of the matrix compartment facilitated the FRET detection.

## Future Developments

Following the first demonstration that the 1,4-benzodiazepine Bz-423 induced apoptosis in living B cells by stimulating superoxid production of the OXPHOS complexes (Blatt et al., 2002) the identification of its molecular target in mitochondria was accomplished by biochemical methods. Subunit OSCP of F_o_F_1_-ATP synthase being the destination of the drug was unraveled by human cDNA T7 phage display screening. Genetic removal of OSCP in mutant F_o_F_1_-ATP synthases proved that apoptosis required binding of Bz-423 to this subunit. Using purified soluble OSCP, the amino acids involved in the binding site were discovered by NMR spectroscopy (Stelzer et al., 2010).

A detailed view on the Bz-423 binding site at the interface of OSCP with the N-termini of α and β subunits of F_1_ is permitted by the recent high-resolution CryoEM structures of the complete mitochondrial enzymes from bovine heart and from yeast *S. cerevisiae* (Zhou et al., 2015; Guo et al., 2017; Srivastava et al., 2018). Both OSCP and the N-termini of α and β subunits change their conformations during catalysis, and Bz-423 binding might interfere with these changes and might retard the turnover. Subsequently the mitochondrial PMF builds up, mitochondria switch from respirational state 3 to 4, superoxide is produced, and Ca^2+^ signaling and apoptosis are initiated. Bz-423 induced the opening of the mitochondrial permeability transition pore and thus decreased the Ca^2+^ retention capability (Giorgio et al., 2013, 2017; Antoniel et al., 2014, 2018; Bernardi et al., 2015).

The weak binding affinities of Bz-423 and its fluorescent derivatives like Bz-423-Cy5 prevented direct fluorescence microscopy approaches in living cells. In initial confocal microscopy experiments we noticed a broad spatial distribution of Bz-423-Cy5 in *S. cerevisiae* cells but not a specific staining of the mitochondria. Therefore we evaluated the use of SMPs with fluorescently tagged F_o_F_1_-ATP synthase for FRET *in vitro* but failed to detect sensitized FRET acceptor emission due to the high fluorescent background of unbound Bz-423-Cy5. The solution for a FRET-based direct detection of Bz-423 binding to OSCP was FRET acceptor photobleaching in mitochondria of living cells. Here the pool of unbound Bz-423-Cy5 is limited, and photobleaching Cy5 with 640 nm at high power is possible without destroying the FRET donor fluorophore yEGFP on F_o_F_1_-ATP synthase. Now, similar FRET experiments are under way with human HEK cells and by using the brightest and more photostable green fluorescent protein mNeonGreen (Shaner et al., 2013) fused to the C-terminus of the γ subunit of F_o_F_1_-ATP synthase (Foertsch et al., 2017).

To improve specificity of the cellular distribution of the hydrophobic Bz-423 derivatives and to accelerate an uptake into mitochondria, attaching cationic dyes could be used. As shown by Irion et al. (1993), Rottele and Zimmermann (1993), Schneider and Zimmermann (1994), and Schneider et al. (1994), almost all fluorophores being lipophilic cations transfer quickly to the inner mitochondrial membrane. There, they can bind to proteins. One identified target was cytochrome C oxidase. This finding could be used to specifically induce photodamage by singlet oxygen as a ROS (Cernay and Zimmermann, 1996; Dummin et al., 1997; Borsch, 2010) for Photodynamic Therapy. Beside photoaffinity labeling approaches, time-resolved FRET was applied to reveal that cytochrome C oxidase was a binding site of these lipophilic cationic photosensitizers acting as FRET donors (Huglin et al., 1995; Borsch, 2010). Similarly, confocal imaging FRET donor lifetimes of mitochondrial F_o_F_1_-ATP synthases in the presence and absence of FRET acceptor-tagged Bz-423 derivatives could be employed to provide direct optical evidence for Bz-423 binding to F_o_F_1_-ATP synthase. Alternatively, a photoaffinity tag on Bz-423 might be necessary to detect binding of the drug to the multimeric form of the enzyme.

Förster resonance energy transfer-based direct evidence for Bz-423 acting at the mitochondrial F_o_F_1_-ATP synthase needs to be complemented by detailed analysis of cristae morphology changes. In mitochondria of living cells, this can be accomplished by superresolution microscopy approaches, for example SIM (Shao et al., 2011) or STED (Schmidt et al., 2009) microscopy. Especially, a rearrangement of row assembly of dimeric F_o_F_1_-ATP synthases at the rim of the cristae could indicate the beginning and early events of apoptosis. Finally, an unequivocal mechanistic demonstration of how Bz-423 affects the catalytic activity and the rotary motors of mitochondrial F_o_F_1_-ATP synthase is awaited. Single-molecule FRET between different fluorophores attached to rotor and stator of the mitochondrial enzyme has already been developed. Accordingly, the inhibition mechanism of Bz-423 could be unraveled by single-molecule rotation experiments, as shown previously for the inhibitor Aurovertin (Johnson et al., 2009) or of phytopolyphenols (Sekiya et al., 2017).

## Author Contributions

All authors designed the experiments and provided chemicals, biological materials and microscopy tools. All authors wrote the manuscript and approved it for publication.

## Conflict of Interest Statement

The authors declare that the research was conducted in the absence of any commercial or financial relationships that could be construed as a potential conflict of interest.
